# Mechanical Performance and Void Structure Change of Foamed Cement Paste Subjected to Static and Cyclic Loading under Plane Strain Conditions

**DOI:** 10.3390/ma15051711

**Published:** 2022-02-24

**Authors:** Zhen Zhang, Fengrui Rao, Guanbao Ye, Jiangting Liu

**Affiliations:** 1 Key Laboratory of Geotechnical and Underground Engineering of Ministry of Education, Department of Geotechnical Engineering, Tongji University, Shanghai 200092, China; dyzhangzhen@gmail.com (Z.Z.); 18829037940@163.com (J.L.); 2 Shanghai Construction No. 2 (Group) Co., Ltd., Shanghai 200090, China; 1710019@tongji.edu.cn

**Keywords:** foamed cement paste, compressive strength, void structure, plane strain condition, cyclic loading

## Abstract

Cement-based lightweight materials have received much attention recently in embankment backfill applications, the boundary of which is more close to a plane strain condition. To study the influence of plane strain condition on the behavior and void structure of cement-based lightweight material under cyclic loading, this paper conducted a series of compression tests on foamed cement pastes with densities of 700 and 900 kg/m^3^ subjected to static and cyclic loading under plane strain conditions. The X-CT technique was adopted to obtain the three-dimensional (3-D) void structures of the specimens before and after the loading tests. The results showed that the plane strain conditions yielded specimen compression strengths 30–50% higher than the unconfined conditions. The specimen integrity endured under load levels of less than 0.5, but failed after approximately 1000 cycles under a load level of 0.8, indicating that cyclic loading could accelerate the degradation of the specimena. The void structures of the specimens showed that the void volumes were featured bfatured an unimodal distribution with unimodal positions in a range of 0.1–0.2 mm^3^. The unimodal position became higher with the increasing cyclic load level. Slices of the specimens after static and cyclic loading tests suggested that cyclic load could easily lead to the rupture of voids that then merge into bigger voids and the connection of voids forming cracks.

## 1. Introduction

Cement-based lightweight materials, such as foamed concretes and cement mortars, which are made of foam, water and cement with or without fine sand, have advantages of adjustable density and strength [1], outstanding performance [2,3] and convenience for construction in-place [4]. Therefore, they have been increasingly used in engineering applications, such as embankments [5], bridge approaches [6], precast walls [7], aircraft arresting system [8], and insulation floor/roof screeds [9].

The cement-based lightweight materials have unique porous structures and engineering characteristics. Previous studies showed that the compressive strength of foamed concrete was related to its density [10], filler type [11], foaming agent [12] and curing conditions [13]. However, previous studies were mostly based on the unconfined compression tests. The stress boundary of foamed concrete in embankment and/or trench backfills is closer to a plane strain condition, as in such conditions, one dimension is very large compared to the others [14,15,16], which could influence its mechanical properties [17,18], but few studies have considered yet the influence of plane strain conditions. The findings based on the unconfined compression tests may not be suitable for evaluating the mechanical properties of foamed concrete under plane strain conditions. On the other hand, the previous studies have mainly focused on the behavior of foamed concrete under static loading. Foamed concrete is sometimes subjected to a cyclic loading (e.g., traffic loads or machine vibration loads) [19,20,21]. Huang et al. [19] proposed a theoretical expression for the fatigue life of foamed cement under uniaxial compression, but the effects of loading frequency and stress ratio were not considered in the study. Huang et al. [20] demonstrated that dynamic loads would weaken the strength of foamed concrete as compared with static loads through flexural experiments, but the study lacked compression test investigations. As a result, further study is needed on the dynamic behavior of foamed concrete under plane strain conditions.

Voids in foamed concrete affect its mechanical behavior. Kearsley and Wainwright [22] pointed out that the compressive strength decreased exponentially with a reduction in the dry density of the foamed concrete. Nambiar and Ramamurthy [23] found that the size of voids increased sharply with an increase in the foam volume thus weakening the strength. With the help of advanced apparatus, such as optical microscopy [24], mercury intrusion porosimetry [25], scanning electron microscopy (SEM) [26] or X-ray computed tomography (X-CT) [27], the investigations deepen into the mesoscale. The results showed that the void size, void spacing, void shape, as well as their distributions would affect the mechanical behavior of foamed concrete [11]. Previous studies generally captured the void structure of foamed concrete before loading, while paying less attention to the changes of void structure after loading tests. Sun et al. [28] investigated the development of voids in foamed concrete by installing a small loading device into a medical X-CT scanner. However, this study only provided the variation of porosity of the foamed concrete, but the void structure was not obtained from the experiments. Some numerical studies were conducted to depict the variation of void structures during loading processes [29,30,31,32], Nguyen et al. [30] analyzed the evolution of void connectivity during compression using a finite element method. This study only provided a phenomenological description of the void connectivity, and lacked data about the variations of void size or void shape, which would affect the behavior of foamed concrete as mentioned previously. Therefore, further studies are necessary to investigate the changes of the void structures of foamed concrete caused by static or cyclic loading.

This paper investigated the mechanical behaviors and void structures of foamed cement paste subjected to static and cyclic loading under plane strain conditions. The X-CT scanning and image-processing techniques were applied to obtain the mesoscopic three-dimensional (3-D) void structures (including the void volume and shape, the void spacing as well as distributions). A statistical analysis was then conducted to analyze the development of the void structure during loading processes.

## 2. Materials and Methods

### 2.1. Preparation of Specimens

This study used a foamed cement paste made of cement, foam and water. Ordinary Portland cement with 28-day unconfined compression strength of 32.5 MPa and tap-water were used. This study used a commercially available synthetic foaming agent (Southern Chemical, Dezhou, China) with sodium lauryl sulfate as the main component. This is an anionic surfactant, commonly used in industrial foam production [33,34]. The performance indices of this foaming agent given by the manufacturer are as follows: powder specific gravity of 1.1, expansion ratio of 50, pH value of 6.5 to 7.5, one-hour settlement less than 70 mm, and foam density of 70 kg/m^3^.

A proper water/cement ratio, which is defined as a weight ratio of water to dry cement, should be determined to ensure the cement mix has adequate workability (i.e., good flowability and good fusion with foams). In this work, foamed concrete mixes with different water/cement ratios (0.3–0.8) were made, and their workability was quantified by the spreadability test using a metal cylinder with 80 mm diameter and 80 mm height. The desired spreadability value was in a range of 160 to 180 mm. The trial tests demonstrated that a water-cement ratio of 0.5 was optimum for this study, which is also in consistent with previous studies [35,36]. The stability of the foam is evaluated by a 1 h settlement test and a 1 h liquid bleeding test. The foam was put in a measuring cup with an inner diameter of 108 mm, a net height of 108 mm and a wall thickness of 2 mm. After 1 h, the settlement and the liquid bleeding were measured. The tests were conducted at a temperature of 25 °C. In this study, the 1 h settlement and 1 h liquid bleeding of foam results were 62–65 mm and 12–13 mL, respectively. The foam was produced using the dry method, where foam is produced by forcing the foaming agent solution and compressed air into a mixing chamber and then ejecting them. Then the foam is blended into the cement paste. The previous studies indicated that this method can produce good-quality foamed concrete as the bubbles produced have uniform sizes and are stiff and stable in the foamed concrete paste [4,13].

Foamed concrete typically has a density of 300–1600 kg/m^3^, however, foamed concrete with a density of 500–1000 kg/m^3^ is commonly used in practice [4,5]. Thus, densities 700 and 900 kg/m^3^ were selected in this study (labelled as FC7 and FC9, respetively). The mix proportion for each density was: (1) FC7: $m_{w}/m_{c}=0.5$, $V_{\mathrm{foam}}/V_{w}=3.5$; (2) FC9: $m_{w}/m_{c}=0.5$, $V_{\mathrm{foam}}/V_{w}=1.5$, where *m*_w_/*m*_c_ = water-cement ratio, which was taken as 0.5 in this study; *V*_foam_/*V*_w_ = foam volume ratio, defined as a ratio of foam volume to water volume. The specimens required a density deviation less than 5% in the same group. The prepared specimens were 40 mm in side length and 80 mm in height. All the specimens were cured in a curing room for 60 days at a temperature 25 ± 1 °C and humidity greater than 95% before testing.

### 2.2. Test Setup and Instrumentation

**Figure 1:**
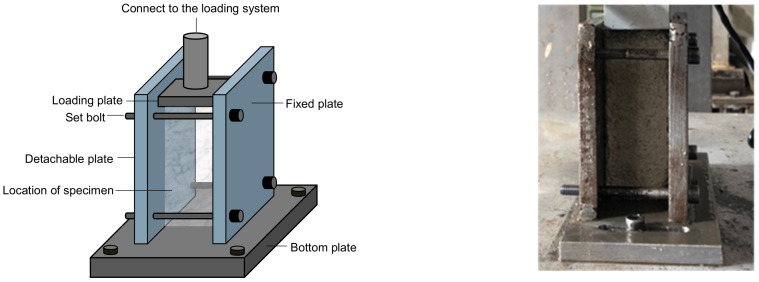
The device to achieve the plane strain condition during the compression test.

**Figure 2:**
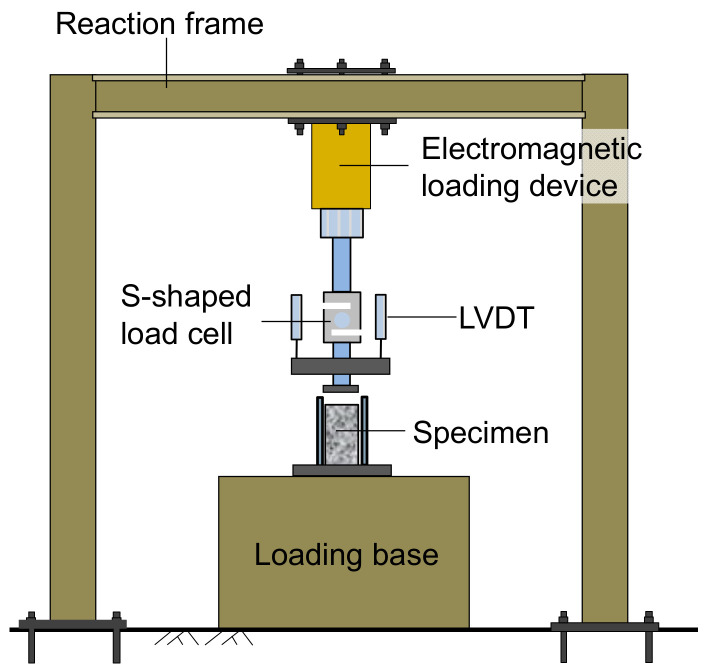
Schematic of plane strain cyclic compression apparatus.

Plane strain conditions are encountered in some practical uses of foamed concrete, such as embankment backfill or cast-in-place walls, where long structures with a constant cross-section and with loading in the plane of the cross-section is a concern [16]. This study designed a device to perform compression tests under plane strain conditions. Figure 1 shows the device fixed on the loading base. Two steel plates were vertically connected to the bottom plate, thus only allowing the lateral deformation of a specimen in one direction and restricting its lateral deformation in the other direction. One plate was fixed to the bottom plate, and the other was movable. After the specimen was placed between the two plates, the removable plate was adjusted to just touch the specimen. A thin layer of Vaseline was smeared on the inside of the two plates to reduce the friction between the specimen and the plate sides. The removable plate was securely fixed with bolts mounted near the four corners of the fixed plate. An electromagnetic loading device was connected to the loading plate, applying a static or a cyclic load. The applied load was monitored using an S-shaped load cell mounted to the loading plate. Four linear variable differential transducers (LVDTs) were installed above the loading plate to monitor the vertical deformation of the specimen during loading, as shown in Figure 2.

### 2.3. Compression Test

The plane strain compression tests under static loading were performed at a displacement rate of 0.2 mm/min until specimen failure. For the sake of comparison, unconfined compression tests were also conducted under the same displacement rate. After the plane strain compression strength of a specimen was determined from the test, a series of compression tests under static loading with load levels of 0.2, 0.5 and 0.8 were conducted. The load level, denoted as *R*, is a ratio of the applied stress to the plane strain compression strength of the specimen of the same density.

The cyclic loading adopted a square wave load at a load frequency of 2 Hz considering the typical range of load frequency in transportation [37,38,39]. The dynamic load had load levels of 0.2, 0.5 and 0.8, the same as the static loading tests. The cyclic loading tests were terminated at either 10^4^ cycles or specimen failure, whichever occurred first.

### 2.4. X-CT Scanning

The X-CT technique has been increasingly used to inspect the internal micro-defects of a product without any destruction to a specimen [28,32,40]. This study adopted a METROTOM-800 CT scanner (MSI Viking, Duncan, CA, USA) to examine the internal structures of the specimens before and after the loading tests. The scanning interval of 32 μm was adopted and 2500 CT slices were obtained for each specimen. The voltage and current applied in the X-ray tube were 160 kV and 0.16 mA, respectively. The X-ray focus size was 1 μm and its view field was scaled by 2048 × 2048 pixels. The X-CT scanning could cover voids of almost all sizes in the specimen with precision less than 40 μm.

To prevent beam hardening during the scanning process [40], 1 mm thick copper was placed between the source and specimen to filter out lower energies. As a result, a longer acquisition time is needed for compensation due to a lower signal-to-noise ratio. Non-local means filtering [41] was used in denoising process for its advantage of preserving both edges and textures. The segmentation of cement matrix and air-voids in images was conducted based on gray-level. All pixels with gray-level lower (or greater) than a threshold were then assigned to one class (or the other class). In order to ensure reproducibility and user-independence, the threshold was selected automatically by the software VGStudio Max (Version 3.2, Beijing, China) installed on the METROTOM-800.

**Figure 3:**
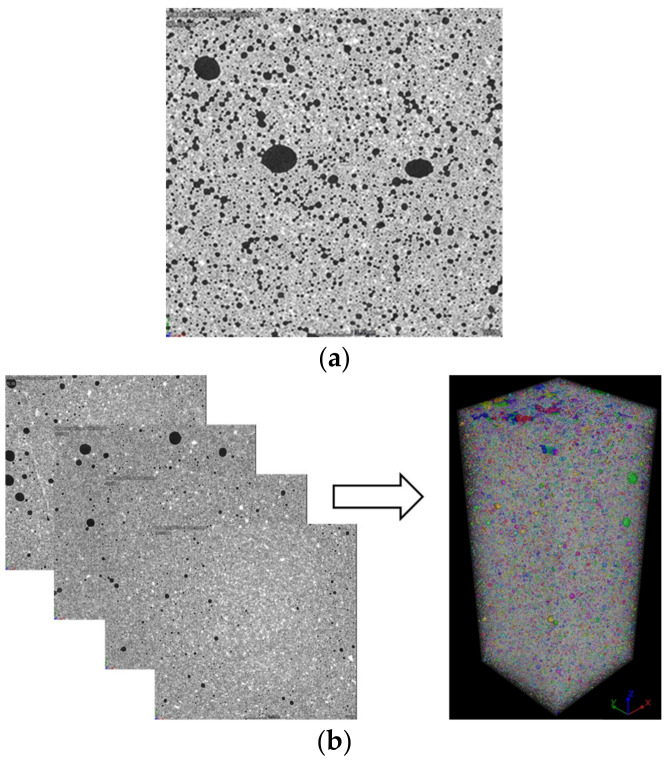
X-CT images of test specimen: (**a**) 2-D image, (**b**) 3-D reconstructed model.

Figure 3a shows a typical X-CT cross-sectional image of a tested specimen in greyscale. The greyscale areas reflect the absorptions of the X-ray for components of different densities. The higher greyscale (dark color) represents the components with lower density, i.e., the voids in the specimen, and the grey and/or white colors represent the component with a larger density, i.e., the hardened cement. Sequentially, 3-D models were reconstructed by stacking a series of 2-D slices (see Figure 3b). Based on the image processing, the void information can be obtained, such as void location, void volume, surface area and void spacing. For ease to distinguish between the voids and the cement skeleton under the 3-D condition, the voids were refilled with random colors.

### 2.5. Test Procedure

The following test procedure was adopted in this study:

(1) Prepare the specimens for each target density (i.e., of 700 and 900 kg/m^3^);
(2) Cure all the specimens under a standard condition with an ambient temperature of 25 ± 1 °C and humidity greater than 95% for 60 days after demolding;
(3) Scan the specimens by X-CT, establish the corresponding 3-D void structural models of the specimens and obtain the void information;
(4) Carry out the plane strain compression tests under static and cyclic loading conditions to obtain their mechanical properties; each test group contains six specimens;
(5) After the compression test, scan the specimens by X-CT immediately and obtain the void information.

## 3. Results and Discussion

### 3.1. Compressive Behavior under Static Loading

**Figure 4:**
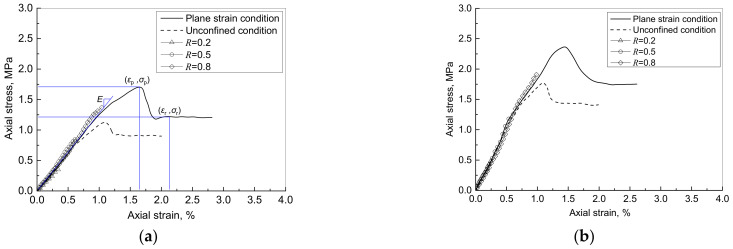
Stress-strain curves of specimen under static loading: (**a**) FC7; (**b**) FC9.

Figure 4 shows the typical stress-strain curves of the foamed cement paste under static loading, including the tests under plane strain conditions and unconfined conditions. Regardless of the lateral boundary condition, the stress increased rapidly with the increase of the strain during the initial stage. After reaching the peak stress, the stress decreased sharply with a small increase of the strain. Next the stress almost remained constant, which is called the residual strength, when the strain continuously increased. In addition, the specimen of FC9 behaved more like a brittle material than the specimen of FC7, as the strain of peak stress of FC9 was less than that of FC7. The typical test results of the plane strain compression test under the loading levels of *R* = 0.2, 0.5 and 0.8 are also included in Figure 4. It is noted that the test results under different loading levels agreed well with the corresponding part of the whole stress-strain curves of the specimen.

The boundary condition has a significant impact on the strength of the foamed cement paste. As compared with the results under the unconfined condition, the plane strain conditions led to an increase in the peak strength of approximately 50%, an increase in the residual strength of approximately 33%, and an increase of the strain under the peak strength of approximately 33% for the specimen of FC7, whereas those for the specimen of FC9 were only approximately 28%, 18%, and 20%, respectively. The less increase of the strength in the specimen of higher density could be explained by the fact that the more brittle material has a lower Poisson’s ratio [42,43].

Some mechanical properties can be determined from the stress-strain curves, including tangent modulus (*E*), peak stress (*σ*_p_) and its corresponding strain (*ε*_p_), and residual strength (*σ*_r_) and its corresponding strain (*ε*_r_). Table 1 shows the properties of all specimens under the plane strain compression test. It can be seen that for the specimens in the same group, the deviations of mechanical properties were larger than density. The coefficients of variation (COV) in the mechanical properties were in a range of 1.45% to 3.43%, whereas the COV of density ranged from 0.06% to 0.11%.

### 3.2. Compressive Behavior under Cyclic Loading

**Figure 5:**
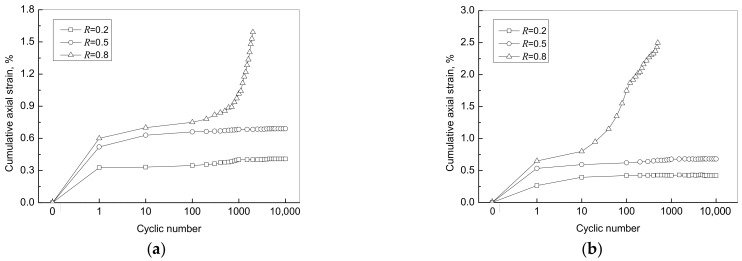
Variation of accumulative axial strain of specimen with cyclic number: (**a**) FC7; (**b**) FC9.

Figure 5 shows the variation of the cumulative axial strain of specimens with the number of cycles under different load levels of 0.2, 0.5 and 0.8. The cumulative axial strain of the specimens of FC7 and FC9 gradually approached a constant value under lower load levels (i.e., *R* = 0.2 and 0.5). However, under the load level of 0.8, the specimens of FC7 and FC9 failed after approximately 1200 and 500 cycles, respectively, following a rapid increase of axial strain. De Andrade et al. [44] and Zhou et al. [45] obtained similar findings in that the lightweight cement composites did not fatigue up to 10^6^ cycles when the applied stress was less than 50% of the peak stress, whereas they could fail below 10^3^ cycles beyond that load level. The studies conducted by Gao et al. [46] and Khajeh et al. [21] also indicated that failure of geo-foam materials was observed after the number of cycles exceeds 10^3^ and is less than 10^4^. Accordingly, it is recommended that the load level be less than 0.5 for the application of foamed cement paste under cyclic loading (e.g., used as backfill material for abutments and embankments) since millions of traffic loads can be expected once in service.

### 3.3. Change of the Void Structure under Static Loading

As the specimens were scanned by the X-CT before and immediately after the loading test, a statistical analysis was conducted to study the change of void structure due to the compression tests, including the void volume and shape, the void spacing as well as their distributions. As indicated in Table 1, the maximum COV in the mechanical properties of the specimens was 3.43%. The previous studies presented that the void structure has an impact on the mechanical properties of foamed concrete [1,10,31]. Even though, considering the slight difference in the mechanical properties of the specimens before loading and the relatively significant change in the void structure due to loading, this study neglects the differences in the intrinsic void structures among the specimens before loading in the same group.

**Figure 6:**
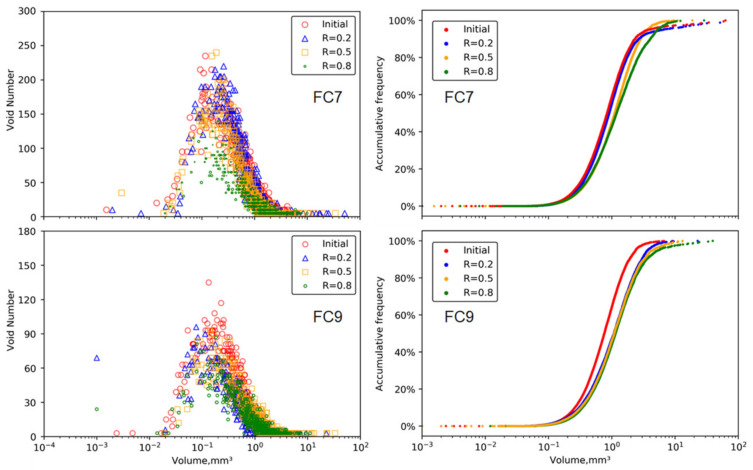
Change of void volume under plane strain quasi-static compression loading.

Figure 6 shows the distributions of the void volumes in both groups, where voids sizes in the range of 0.001–60 mm^3^ were considered for the analysis. Generally, the void volumes had a unimodal distribution with a unimodal position ranging 0.1–0.2 mm^3^, and the number of voids on the left side of the peak was far less than that on the right side. As the load level increased, the distribution of the number of voids kept the same shape but had a lower unimodal position and narrower range, indicating that the total number of voids decreased. However, the curves of accumulative volume percentage indicated that the compressive stress generated more large-sized voids.

Table 2 shows the statistic results of the voids with different types of contact in each group. Based on the measured 3-D internal void structure of the foamed cement paste by the X-CT scanning, a void in a specimen had three types of contact with other voids: (1) surrounded (i.e., the void is surrounded by two or more voids); (2) isolated (a single void is separated from others); (3) edged (the void is at the edge and connected with one void). The software used the wall thickness as the physical criterion to define different types of voids. The void having a wall thickness of 0.01 to 0.1 mm with other voids is defined as border-type void, while the void having a wall thickness larger than 0.1 mm with other voids is defined as an isolated-type void. It can be seen that in both groups, the surrounded-type voids were in the majority by the void volume under all loading levels. As the load level increased, the total volume proportion of surrounded-type voids significantly decreased, while the volume proportions of isolated-type and edged-type voids increased.

**Figure 7:**
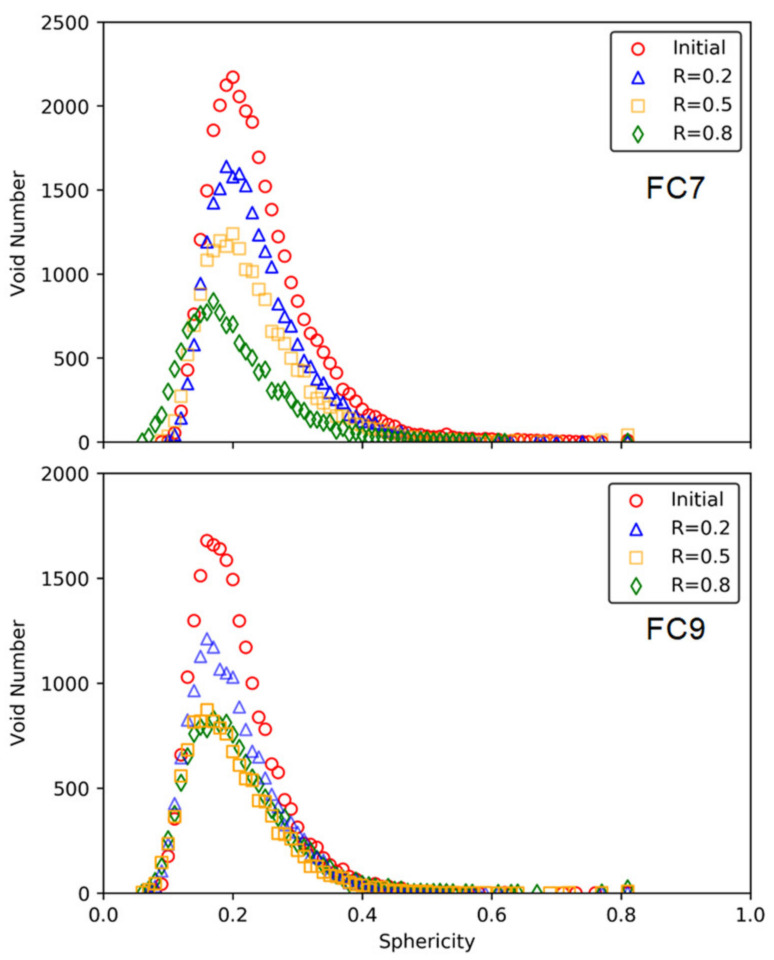
Variation of void sphericity after static loading.

Figure 7 shows the distributions of the void sphericity in each group. The sphericity is an indicator for void shape, which is defined as a ratio of the surface area of a sphere with the same volume of the given void to the surface area of the void. It can be seen that the voids in the foamed cement pastes were highly irregular in shape as the sphericity values were far less than 1.0, mainly in a range of 0.1 to 0.7. Similar findings have been reported in the work of Chung et al. [27]. This distribution indicates that most voids in the foamed cement paste were highly irregular and not close to spheres in shape. As the loading level increased, the distribution retained the unimodal shape with gradually decreased height; however, with the increase of the load level, the median value of void sphericity decreased slightly from 0.22 to 0.19 in the group FC7 and remined at 0.19 in the group FC9. The static compression load had minor impact on void sphericity.

### 3.4. Change of the Void Structure under Cyclic Loading

**Figure 8:**
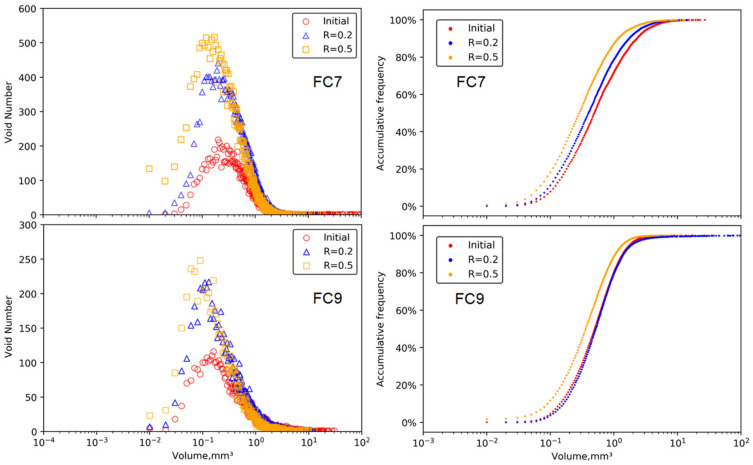
Change of void volume under plane strain cyclic compression loading.

This section presents the results of the specimens under the load levels of 0.2 and 0.5, as the specimens under a load level of 0.8 failed during the loading test. Figure 8 shows the distributions of the void volumes in each group. Similar to the results in the static loading tests, the void volumes featured an unimodal distribution with unimodal positions in a range of 0.1–0.2 mm^3^. The cyclic loading did not change the distribution pattern of the void volume, but different from the distribution under static load, the unimodal position became higher with the increase in load level. This indicates that the total number of voids increased, and that could be further observed from the accumulative volume percentage curves.

Table 3 shows the distributions of the voids with different types of contact in each group. Similar to the results in the static load, with the increase of the load level, the total volume proportion of surrounded-type voids decreased, while the volume proportions of isolated-type and edged-type voids increased. Furthermore, the changes of volume proportion of surrounded-type voids and isolated-type voids became more significant than the corresponding changes due to the static load.

**Figure 9:**
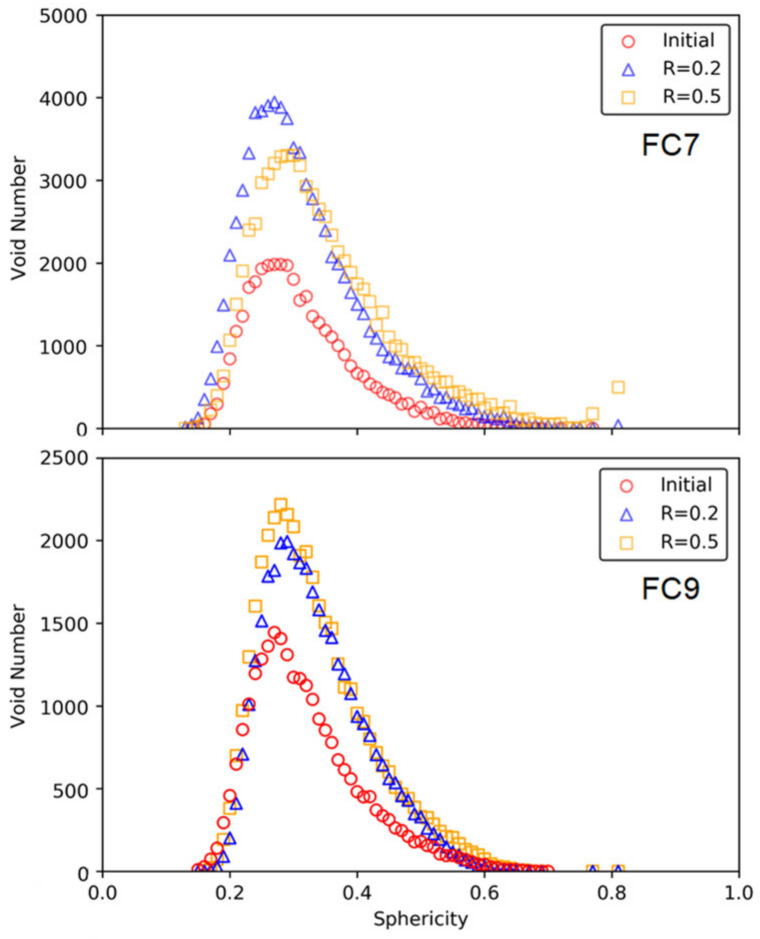
Change of void volume under plane strain cyclic compression loading.

Figure 9 shows the distributions of the void sphericity in each group. The distribution was also characterized by unimodal formation. With the increase of the loading level, the distribution curves retained the same pattern with higher unimodal position, and the void sphericity at the unimodal position was almost maintained unchanged in both group. The cyclic compression load had minor impact on void sphericity. With the increase of the load level, the median value of void sphericity increased slightly in both groups (i.e., from 0.22 to 0.23 in the group FC7, and from 0.25 to 0.26 in the group FC9).

### 3.5. Static Loading versus Cyclic Loading

This section conducts a further study to compare the mechanical behavior of foamed cement paste as well as its void structure under static load and cyclic load.

#### 3.5.1. Deformation

To compare the deformation of foamed cement paste under cyclic and static loading, the dynamic increase factor (DIF) is introduced according to Feng et al. [47]:

(1) $$\mathrm{DIF}=\frac{\varepsilon_{d}}{\varepsilon_{s}},$$

where *ε*_d_ is the constant strain under cyclic loading, and *ε*_s_ is the final strain under the static load with the same magnitude of cyclic load.

Table 4 summarizes the DIF of deformation under the load level of *R* = 0.2 and *R* = 0.5. As expected, cyclic load caused greater deformations than static loads. This could be explained by the reason that cyclic loads have more energy than static loads, resulting in more voids or flaws in the specimen compressed to absorb the impact energy. The DIF decreased with the increase of load level in both group due to the relatively large modulus of foamed cement paste under small load level. The DIF in the group FC9 was larger than that of FC7. The voids could mitigate the cyclic load impact. The specimens of relatively large density as the group FC9 resulted in more impact energy absorbed by the brittle deformation of cement skeleton, thus leading to a large dynamic deformation. Similar conclusion has been reported in the investigation of foamed concrete’s deformation under low-velocity impact [48].

#### 3.5.2. Void Structure

**Figure 10:**
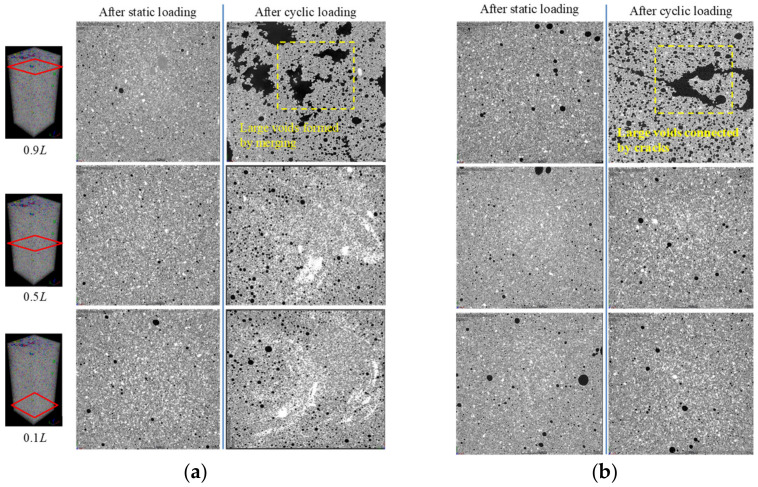
Comparison of cross-sectional greyscales of the FC7 specimens after static and cyclic loading tests: (**a**) *R* = 0.2, (**b**) *R* = 0.5.

**Figure 11:**
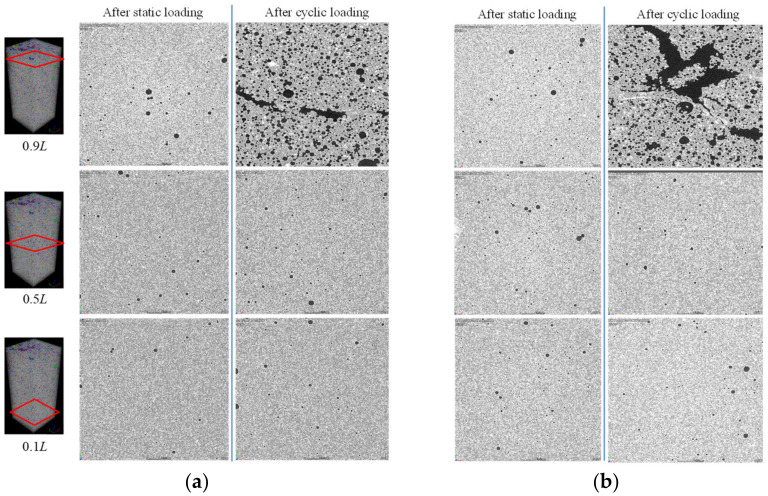
Comparison of cross-sectional greyscales of the FC9 specimens after static and cyclic loading tests: (**a**) *R* = 0.2, (**b**) *R* = 0.5.

For a better presentation, cross-sectional greyscales of the specimens at the locations of 0.1 *L*, 0.5 *L*, 0.9 *L* were selected, where *L* denotes the height of specimen and is 80 mm. Figure 10 shows the cross-sectional greyscales of the specimens in the group FC7 at load levels of 0.2 and 0.5. It can be noted visually that there were no obvious cracks for the specimens under static loading. However, under the same level of cyclic load, some voids at the top portion ruptured, merging into larger voids. Nguyen et al. [30] observed a similar process of void merging of foamed concrete when the static peak strength was reached. Moreover, after adding the cyclic loads with a load level of 0.5, besides the ruptured voids merging into bigger ones, cracks connecting the bigger voids appeared. The damage to the void structures can also be observed in Figure 11, which shows the cross-sectional greyscales of the specimens in the group FC9 at load levels of 0.2 and 0.5. This further explains the fact that the cyclic loads cause more damage to the foamed cement paste as compared with static loads. Furthermore, it could be observed that the occurrence of cracks was concentrated on the top regions in the specimens, indicating that the impact energy had more influence in these top regions.

## 4. Conclusions

This paper has analyzed the mechanical performance and void structure change of foamed cement pastes subjected to static and cyclic loading under plane strain conditions. Based on the analyses and discussion, the following conclusions can be drawn:

1. The compression strengths of foamed cement paste under plane strain conditions was 30–50% higher than under unconfined conditions. This improvement due to the plane strain conditions in the group of 700 kg/m^3^ was more significant than that observed in the group of 900 kg/m^3^.
2. The specimens retained their integrity under cyclic loading when the load level was less than 0.5, but failed after approximately 1000 cycles under a load level of 0.8, indicating that cyclic loading could accelerate the degradation of foamed cement paste. The deformation of specimens under cyclic loading was 10%-60% higher than that under static loading, and the increase in deformation was more significant in the group of 900 kg/m^3^.
3. The total number of voids decreased under static loading; by contrast, it increased under cyclic loading. As the load level increased, the total volume proportion of surrounded-type voids decreased significantly, while the volume proportions of isolated-type and edged-type voids increased. The load condition (static or cyclic load) had minor impact on the median value of void sphericity. Cyclic loads could easily lead to rupturing the voids, merging some voids into bigger ones, and forming cracks.

Although the conclusions were drawn from a 100% foamed cement paste, the results are also beneficial to the knowledge of the foamed concretes obtained by adding aggregate, fine sand and other additives. It will be interesting if the obtained results in this study can be verified by the use of foamed concretes with additives, including fine sand and fly ash in the future.

## Figures and Tables

**Table 1:** Mechanical and physical properties of specimens.

Specimen	Density (kg/m^3^)	*E* (MPa)	*σ*_p_ (MPa)	*ε*_p_ (%)	*σ*_r_ (MPa)	*ε*_r_ (%)
FC7-1	698.8	132.0	1.73	1.70	1.20	1.94
FC7-2	699.5	128.8	1.68	1.76	1.26	1.87
FC7-3	700.3	126.7	1.65	1.64	1.18	1.96
COV(%)	0.11	2.07	2.40	3.53	3.43	2.46
FC9-1	899.6	209.3	2.38	1.48	1.76	2.39
FC9-2	900.2	207.6	2.29	1.44	1.87	2.32
FC9-3	900.7	213.5	2.33	1.52	1.82	2.46
COV(%)	0.06	1.45	1.93	2.70	3.03	2.93

Note: COV = Coefficient of variation.

**Table 2:** Volume proportions of voids in different contact relationship under static loading.

Group	Load Level	Surrounded (%)	Isolated (%)	Edged (%)
FC7	Initial	78.404	0.307	21.289
FC7	0.2	74.355	1.548	30.097
FC7	0.5	70.217	2.233	36.550
FC7	0.8	63.872	3.326	40.802
FC9	Initial	73.235	1.190	25.575
FC9	0.2	68.667	1.408	32.741
FC9	0.5	64.149	1.654	34.197
FC9	0.8	60.383	1.854	37.763

**Table 3:** Volume proportion of voids in different contact relationship under cyclic loading.

Group	Load Level	Surrounded (%)	Isolated (%)	Edged (%)
FC7	Initial	78.404	0.307	21.289
FC7	0.2	75.333	4.273	20.394
FC7	0.5	67.258	9.667	23.075
FC9	Initial	73.235	1.190	25.575
FC9	0.2	63.326	7.587	29.087
FC9	0.5	55.167	12.259	32.574

**Table 4:** DIF of deformation.

Group	Load Level	DIF
FC7	0.2	1.234
FC7	0.5	1.129
FC9	0.2	1.619
FC9	0.5	1.273

## Data Availability

The data that support the findings of this study are available from the corresponding author, Guanbao Ye, upon reasonable request.
